# Post-VABB Cavity-Targeted Avidin-Pretargeted [^90^Y]Y-DOTA-Biotin in Nonpalpable Breast Cancer: A Phase I Activity-Escalation Study (ARTHE)

**DOI:** 10.3390/cancers18172760

**Published:** 2026-08-25

**Authors:** Maddalena Sansovini, Paola Sanna, Paola Possanzini, Emanuela Scarpi, Irene Marini, Silvia Nicolini, Ilaria Grassi, Paola Caroli, Michele Amadori, Annalisa Curcio, Giulia Simoncini, Lucia Fabbri, Oriana Nanni, Manuela Monti, Lilla Vizza, Anna Miserocchi, Valentina Di Iorio, Cristina Cuni, Maria Luisa Belli, Matteo Costantini, Fabio Falcini, Giovanni Paganelli, Federica Matteucci, Anna Sarnelli

**Affiliations:** 1 IRCCS Istituto Romagnolo per lo Studio dei Tumori (IRST) “Dino Amadori”, 47121 Meldola, Italy; maddalena.sansovini@irst.emr.it (M.S.); emanuela.scarpi@irst.emr.it (E.S.); irene.marini@irst.emr.it (I.M.); silvia.nicolini@irst.emr.it (S.N.); ilaria.grassi@irst.emr.it (I.G.); paola.caroli@irst.emr.it (P.C.); lucia.fabbri@irst.emr.it (L.F.); oriana.nanni@irst.emr.it (O.N.); manuela.monti@irst.emr.it (M.M.); lilla.vizza@irst.emr.it (L.V.); anna.miserocchi@irst.emr.it (A.M.); valentina.diiorio@irst.emr.it (V.D.I.); cristina.cuni@irst.emr.it (C.C.); maria.belli@irst.emr.it (M.L.B.); giovannipaga55@gmail.com (G.P.); anna.sarnelli@irst.emr.it (A.S.); 2 Azienda Unità Sanitaria Locale della Romagna, 47122 Forlì, Italy; paola.sanna@auslromagna.it (P.S.); paola.possanzini@auslromagna.it (P.P.); michele.amadori@auslromagna.it (M.A.); annalisa.curcio@auslromagna.it (A.C.); giulia.simoncini@auslromagna.it (G.S.); matteo.costantini@auslromagna.it (M.C.); fabio.falcini@auslromagna.it (F.F.); 3 Department of Translational Medicine and for Romagna, University of Ferrara, 44121 Ferrara, Italy

**Keywords:** breast cancer, vacuum-assisted breast biopsy, yttrium-90, DOTA-biotin, avidin-mediated local trapping, targeted radionuclide therapy, intralesional therapy

## Abstract

Background/Objectives: Vacuum-assisted breast biopsy (VABB) is widely used for the diagnosis of nonpalpable breast cancer but is not considered definitive treatment because microscopic residual disease may persist within or around the biopsy cavity. The ARTHE phase I trial evaluated a cavity-targeted radionuclide strategy based on same-session sequential intralesional administration of avidin followed by [^90^Y]Y-DOTA-biotin after VABB. Methods: Eighteen women with nonpalpable breast cancer measuring ≤15 mm and a skin-to-cavity distance ≥ 13 mm were treated in three sequential activity-escalation cohorts. The primary objective was to assess acute local and systemic safety, including dose-limiting toxicity. The protocol-specified co-primary objective was to evaluate preliminary antitumor activity, assessed as breast-only pathologic complete response (pCR) at surgery. Given the phase I single-arm design, pCR was analyzed descriptively. The protocol-specified secondary objective was patient-specific dosimetry. Additional exploratory assessments included post-injection biodistribution and integration with subsequent breast-conserving surgery. Results: No dose-limiting toxicities, grade ≥ 3 adverse events, clinically relevant hematologic toxicity, treatment discontinuations, hospitalizations, or treatment-related surgical delays were observed. Local toxicity was limited to grade 1 injection-site pain and/or erythema. Post-injection imaging consistently demonstrated focal intralesional localization without clinically relevant extra-lesional uptake. All patients underwent breast-conserving surgery 4–7 weeks after treatment. No residual tumor cellularity (RTC), from either invasive or in situ carcinoma, was identified in the breast surgical specimen in 5/18 patients (27.8%). However, because diagnostic VABB may have removed part or all of the malignant lesion, the absence of residual carcinoma at surgery cannot be attributed specifically to the radionuclide treatment. Conclusions: The intralesional avidin-mediated local trapping strategy using [^90^Y]Y-DOTA-biotin after VABB was feasible, showed favorable acute and short-term tolerability, was associated with early focal localization on post-injection imaging, and was compatible with standard breast-conserving surgery. Controlled studies are warranted to determine the therapeutic contribution of this post-biopsy cavity-targeted strategy, optimize administered activity, and refine patient selection.

## Simple Summary

Vacuum-assisted breast biopsy is commonly used to diagnose small breast cancers that cannot be felt. Even when the visible lesion appears to have been removed during the biopsy, microscopic tumor tissue may remain around the biopsy cavity, and surgery is therefore still required. The ARTHE phase I trial investigated a novel local radionuclide treatment delivered into the post-biopsy cavity. Eighteen women received intralesional avidin followed by [^90^Y]Y-DOTA-biotin at increasing activity levels. The treatment was well tolerated during short-term follow-up, showed early focal localization within the treated post-VABB region on post-injection imaging, and did not delay or interfere with subsequent breast-conserving surgery. No severe treatment-related adverse events or dose-limiting toxicities occurred. Complete absence of tumor in the breast surgical specimen was observed in five patients, although the contribution of the radionuclide treatment cannot be distinguished from the tissue removed during the biopsy. Further controlled studies are needed.

## 1. Introduction

Nonpalpable breast lesions are increasingly detected through screening programs and high-resolution breast imaging and account for a substantial proportion of abnormalities referred for tissue diagnosis and, when malignant, surgical treatment. Vacuum-assisted breast biopsy (VABB) has become a standard minimally invasive approach for the percutaneous diagnosis of these lesions because of its high diagnostic accuracy, large tissue yield, and favorable safety profile. Its use may also avoid diagnostic surgical excision and intraoperative frozen-section analysis [1,2].

In selected benign or high-risk breast lesions, vacuum-assisted excision may achieve complete lesion removal and can represent an alternative to surgical excision in appropriately selected cases [3,4]. In malignant disease, however, VABB is not currently considered definitive treatment, even when the target lesion appears to have been partially or completely removed during the diagnostic procedure or when post-biopsy imaging shows no residual microcalcifications or detectable lesion [5,6,7,8,9,10]. As a result, a clinically relevant interval remains between percutaneous diagnosis and definitive surgery, during which microscopic residual disease may persist within or around the biopsy cavity.

This interval represents a unique therapeutic window of opportunity because the post-biopsy cavity remains directly accessible before definitive surgery, potentially allowing localized treatment to be delivered to the area at highest risk of harboring microscopic residual disease. Targeted radionuclide therapy is particularly attractive in this setting because it may deliver localized beta-particle irradiation to the cavity wall and surrounding tissue while limiting unnecessary exposure to the remaining breast. Unlike conventional molecularly targeted radionuclide therapies, the target in this approach is primarily anatomical and procedure-defined: the post-VABB cavity and its surrounding tissue. To our knowledge, no previous clinical study has investigated this cavity itself as a therapeutic target for localized radionuclide treatment in patients with nonpalpable breast cancer.

Avidin is a 66 kDa glycosylated, positively charged protein characterized by a high isoelectric point and exceptionally strong affinity for biotin. Previous clinical observations using avidin and [^111^In]In-labeled biotin in inflammatory conditions suggested that avidin-based pretargeting may support localized retention of subsequently administered radiolabeled biotin in biologically altered tissues [11]. On this basis, the inflammatory and tissue-remodeling microenvironment generated by VABB was hypothesized to favor local persistence of avidin within the biopsy cavity.

Avidin–biotin pretargeting has also been investigated for radionuclide treatment of breast cancer, particularly within the IART clinical program. In IART, avidin was administered intraoperatively into and around the surgical tumor bed after breast-conserving surgery, followed 12–24 h later by intravenous administration of radiolabeled biotin [12,13,14]. In contrast, ARTHE investigates a preoperative, post-VABB strategy in which avidin and [^90^Y]Y-DOTA-biotin are administered sequentially by the same intralesional route during a single treatment session. Thus, rather than reproducing conventional systemic pretargeting, ARTHE exploits the post-biopsy target region as an anatomically accessible site for avidin-mediated local trapping of the radiolabeled ligand. This specific same-session intralesional strategy has not previously been evaluated in the post-VABB setting of patients with nonpalpable breast cancer. ARTHE was therefore designed as a single-center phase I activity-escalation trial evaluating an intralesional avidin-mediated local trapping strategy using [^90^Y]Y-DOTA-biotin in the post-VABB target region. The primary objective was to assess acute local and systemic safety and tolerability, including dose-limiting toxicity. The protocol-specified co-primary objective was to evaluate preliminary antitumor activity, assessed as breast-only pCR at surgery. Given the single-arm phase I design, the pathologic endpoint was intended as a descriptive, hypothesis-generating measure rather than as evidence of treatment efficacy. The protocol-specified secondary objective was patient-specific dosimetry. Additional exploratory assessments included post-injection biodistribution and integration with subsequent breast-conserving surgery.

## 2. Materials and Methods

### 2.1. Study Design and Oversight

ARTHE—Avidination for Radionuclide Therapy in Nonpalpable Breast Cancer (EudraCT 2021-000661-33; ClinicalTrials.gov NCT06390241)—was an investigator-initiated, single-center, open-label, single-arm Phase I activity-escalation trial conducted at the IRCCS Istituto Romagnolo per lo Studio dei Tumori (IRST) “Dino Amadori”, Meldola, Italy.

The study evaluated a cavity-targeted radionuclide treatment consisting of same-session sequential ultrasound-guided intralesional administration of avidin followed immediately by [^90^Y]Y-DOTA-biotin, also referred to as r-BHD, into the post-VABB cavity.

The protocol was approved by the Ethics Committee of Romagna (Comitato Etico della Romagna, CEROM) on 9 May 2024, the Italian Medicines Agency (AIFA), and the Italian Ministry of Health for radiation-protection purposes, in accordance with applicable national regulations. The study was conducted in accordance with the Declaration of Helsinki and Good Clinical Practice. All participants provided written informed consent before undergoing any study-specific procedure.

The trial comprised an activity-escalation phase followed by a planned expansion phase at the selected activity level. The present report describes the completed activity-escalation phase. The activity levels of 28, 57, and 126 MBq represented prespecified nominal target activities used for cohort assignment and escalation. Actual administered activity was individually measured for each patient and is reported descriptively by cohort. Differences between nominal and administered activity reflected technical variability related to radiopharmaceutical preparation, dispensing, activity measurement, and timing of administration.

Patients were treated sequentially in three cohorts of six participants each. Escalation to the next activity level was permitted only after completion and review of the prespecified acute safety window for the preceding cohort.

The activity levels were selected on the basis of model-based planning simulations performed before clinical implementation under simplified assumptions, including an approximately spherical biopsy cavity with a volume of 1 mL, uniform activity distribution, and limited activity washout.

The primary objective was to assess acute local and systemic safety and tolerability, including dose-limiting toxicity, and thereby inform selection of an activity level for subsequent clinical development. The protocol-specified co-primary objective was to evaluate preliminary antitumor activity, assessed as breast-only pathologic complete response (pCR) at definitive surgery. Given the single-arm phase I design, the co-primary pathologic endpoint was analyzed descriptively and was not intended to establish treatment efficacy. The protocol-defined dose-limiting toxicity was any grade ≥ 3 cutaneous adverse event occurring within 10 days after intralesional administration and considered by the investigator to be related to the study treatment or administration procedure. All other local and systemic adverse events were prospectively collected and included in the overall safety assessment. The protocol-specified secondary objective was evaluation of patient-specific absorbed dose and dosimetry. Additional exploratory assessments included post-injection biodistribution, clinically relevant off-target localization, feasibility of integration with breast-conserving surgery, and descriptive histologic findings. The trial was conducted within a certified phase I clinical trial unit compliant with AIFA Determination 809/2015 for early-phase nuclear medicine studies. The unit included dedicated facilities for investigational radiopharmaceutical preparation, radiation protection, post-injection imaging, clinical monitoring, and trial data management.

### 2.2. Patient Population

Women aged 18–75 years with nonpalpable breast lesions classified as BI-RADS 4 or 5 on mammography and/or ultrasonography were screened for eligibility. For the purposes of the study, a nonpalpable lesion was defined as a breast lesion not detectable on clinical breast examination and requiring imaging guidance for localization and biopsy. Nonpalpability was therefore determined clinically and was independent of the lesion diameter measured on imaging. Participants were eligible for treatment after histologic confirmation of breast carcinoma if the maximum lesion diameter was ≤15 mm and the minimum skin-to-lesion or post-biopsy skin-to-cavity distance was ≥13 mm.

Additional eligibility criteria included invasive or in situ breast carcinoma of any histologic subtype, an Eastern Cooperative Oncology Group performance status of 0–1, and candidacy for breast-conserving surgery after VABB. Women of childbearing potential were required to use effective contraception during study participation and for at least 4 months after treatment.

Key exclusion criteria included previous surgery or radiotherapy to the ipsilateral breast; non-carcinoma histology, including Paget disease; multifocal or diffuse disease; microcalcifications extending over >15 mm; lesions located <13 mm from the skin or in close proximity to the axilla; pregnancy or breastfeeding; previous exposure to avidin; known allergy to egg proteins or latex; metastatic disease; and uncontrolled comorbidities that could compromise treatment safety or protocol compliance.

The minimum skin-to-target distance was assessed by ultrasonography from the skin surface to the nearest margin of the lesion before VABB and/or to the nearest margin of the post-biopsy cavity before intralesional treatment, as applicable.

### 2.3. Study Procedures and Timing

Patients underwent VABB as part of the standard diagnostic work-up. Tissue specimens were submitted for histopathologic evaluation, with results generally available within 72 h. Women with confirmed carcinoma who met the eligibility criteria were contacted and offered participation in the trial. After written informed consent had been obtained, baseline clinical and laboratory assessments were performed.

Intralesional treatment was administered within the protocol-defined window, generally 7–8 days after diagnostic VABB. This interval allowed histopathologic confirmation and completion of baseline assessments while preserving access to the recently formed post-biopsy cavity. Participants were monitored for acute local and systemic toxicity throughout the prespecified 10-day dose-limiting toxicity window and at subsequent protocol-defined safety visits.

Definitive breast-conserving surgery was scheduled according to routine clinical practice and institutional pathways. No protocol-mandated delay or modification of surgery was required because of the investigational treatment. Surgery was performed 4–7 weeks after intralesional administration, and the resected breast specimen was evaluated for residual invasive and in situ carcinoma, including assessment of RTC and for the protocol-defined breast-only pathologic complete response endpoint.

**Figure 1:**
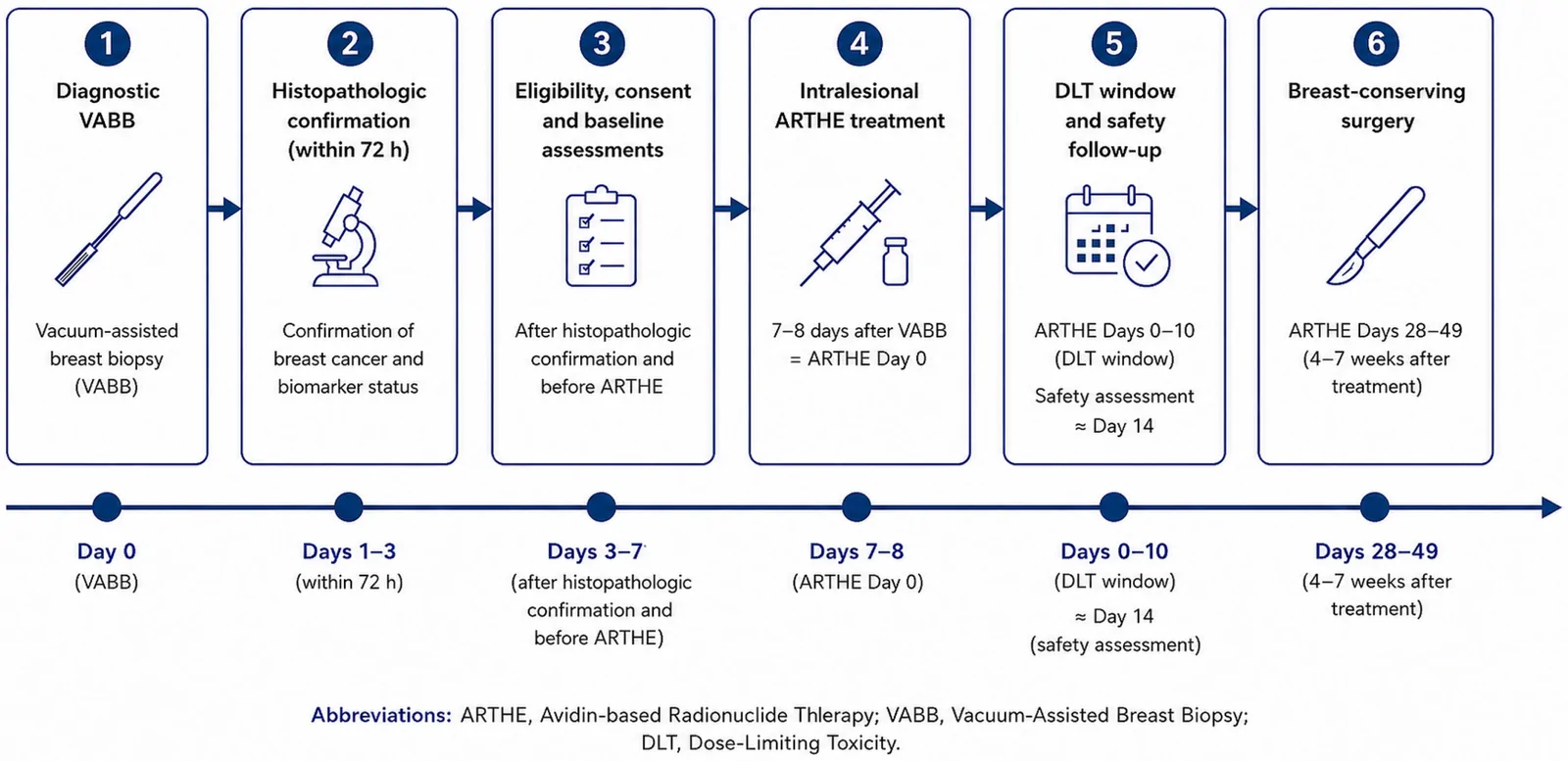
ARTHE trial workflow and timing. Diagnostic VABB was performed on Day 0, with histopathologic confirmation generally available within 72 h, followed by eligibility assessment, informed consent, enrollment, and baseline evaluations. Intralesional ARTHE treatment was administered approximately 7–8 days after VABB and was defined as ARTHE Day 0. The prespecified dose-limiting toxicity window extended from ARTHE Day 0 through Day 10, with subsequent protocol-defined safety assessments, and definitive breast-conserving surgery was performed approximately 28–49 days (4–7 weeks) after ARTHE treatment.

The overall study workflow and timing are summarized in Figure 1.

### 2.4. Vacuum-Assisted Breast Biopsy

VABB was performed under ultrasound or stereotactic guidance using a 9-gauge needle. Excised tissue specimens were immediately fixed in formalin and processed for routine histopathologic evaluation.

At the end of the biopsy procedure, a Q-shaped marker clip was deployed to allow subsequent localization of the biopsy cavity or target region and to guide ultrasound-assisted intralesional treatment, including in lesions initially biopsied under stereotactic guidance. Post-biopsy mammography was performed to verify correct clip positioning.

### 2.5. Investigational Product Preparation

[^90^Y]Y-DOTA-biotin, also referred to as r-BHD, was prepared at the IRST Radiopharmacy in accordance with national requirements governing investigational radiopharmaceutical production. [^90^Y]Y-chloride was supplied by Eckert & Ziegler Radiopharma GmbH, Braunschweig, Germany. The r-BHD ligand was supplied as a good manufacturing practice active pharmaceutical ingredient by ABX GmbH, Radeberg, Germany. Avidin was supplied as an investigational medicinal product by Diatheva S.r.l., 61030 Cartoceto PU, Italy. The biological activity and biotin-binding capacity of avidin had been characterized during manufacturing validation using a colorimetric assay according to Green [15], with predefined acceptance criteria of 11.0–15.0 U/mg for biological activity, >95% active protein/total protein for specific activity, and ≥198 U/mL for biotin-binding capacity.

Radiolabeling was performed aseptically in a shielded isolator by combining r-BHD, a DOTA-conjugated biotin derivative, with [^90^Y]Y-chloride to obtain a specific activity of approximately 3.1–3.2 MBq/μg. The reaction mixture was supplemented with gentisic acid/ascorbic acid buffer at pH 5.0 ± 0.5 and heated at 100 °C for 8 min. The final product was diluted with saline containing ascorbic acid and sterilized by filtration through a 0.22 μm membrane filter.

Quality-control procedures included assessment of radiochemical purity by both high-performance liquid chromatography (HPLC) and thin-layer chromatography (TLC), with a predefined release specification of ≥97% [^90^Y]Y-r-BHD; free [^90^Y]Y and colloidal ^90Y species were each required to be ≤3%. Additional pre-release controls included radiochemical identity, chemical purity, pH, bacterial endotoxins, and filter integrity. Radionuclidic identity and purity of the ^90Y precursor were documented according to the manufacturer’s Certificate of Analysis. Sterility was assessed as a post-release test according to the European Pharmacopoeia. The stability of the final [^90^Y]Y-r-BHD preparation was tested and confirmed for up to 24 h after production. Only preparations meeting all applicable pre-release specifications were administered. Avidin-binding capacity was established during avidin manufacturing validation and was not repeated as a release test on the radiolabeled [^90^Y]Y-r-BHD preparation.

Following quality control, individual patient doses of [^90^Y]Y-DOTA-biotin were aseptically dispensed into 1 mL syringes according to the assigned activity level and adjusted to a fixed injection volume of 0.7 mL with saline.

A nominal avidin dose of 10 mg in 1.0 mL saline was prepared for each patient. Owing to residual volume in the syringe and injection system, the actual administered avidin dose ranged from 6 to 10 mg. This variability was technical and was unrelated to the assigned [^90^Y]Y-DOTA-biotin activity level. The total nominal injection volume was 1.7 mL.

### 2.6. Treatment Administration

Intralesional treatment was administered 7–8 days after VABB in the Nuclear Medicine Department. Under ultrasound guidance, the post-VABB target region was identified on the basis of post-biopsy tissue changes, including altered echogenicity, and/or the position of the marker clip, when visible. A discrete fluid-filled cavity was not required for target localization. Avidin, prepared at a nominal dose of 10 mg in 1.0 mL saline, was injected intralesionally along the margins of the post-VABB target region. Owing to residual volume in the syringe and injection system, the actual administered dose ranged from 6 to 10 mg. Immediately thereafter, [^90^Y]Y-DOTA-biotin was injected into the same target region at the assigned activity level in a fixed volume of 0.7 mL. Patients were observed after administration according to institutional clinical-monitoring and radiation-protection procedures. Follow-up visits were performed according to the protocol to assess acute local and systemic toxicity, including events occurring during the prespecified 10-day dose-limiting toxicity window.

### 2.7. Model-Based Activity Selection and Skin-Distance Constraint

The activity-escalation scheme was established prospectively using model-based planning simulations performed before clinical implementation. The post-biopsy cavity was approximated as a spherical 1 mL volume containing a uniform distribution of [^90^Y]Y-DOTA-biotin activity. Monte Carlo simulations performed using the PENELOPE code (OECD Nuclear Energy Agency; version not available; https://www.oecd.org/en/publications/penelope-a-code-system-for-monte-carlo-simulation-of-electron-and-photon-transport_9d2cc3d5-en.html (accessed on 23 August 2026)) were used to estimate the radial absorbed-dose distribution within and around the cavity as a function of distance from the cavity edge under simplified geometric and kinetic assumptions.

A planning absorbed dose of approximately 200 Gy to potential microscopic residual disease was adopted. This target was informed by previous molecular radionuclide therapy experience, including radioiodine dosimetry concepts used for tissue ablation [16]. However, the 200-Gy value was not based on breast-cancer-specific experimental evidence and should be regarded as a protocol-defined planning assumption rather than as a validated tumoricidal threshold.

Assuming 10% activity washout from the modeled source region, nominal activities of 28, 57, and 126 MBq were estimated to deliver 200 Gy at 1, 2, and 3 mm from the cavity edge, respectively. In the absence of the assumed washout, the corresponding prescription point would receive approximately 222 Gy. Thus, 126 MBq represented the highest prespecified activity because it was the activity required by the planning model to extend the 200-Gy prescription to 3 mm beyond the cavity margin; it should not be interpreted as a maximum tolerated or therapeutically optimal activity.

Skin exposure was evaluated separately using the dose profile corresponding to the highest investigated activity level. The modeled dose fall-off toward the skin was converted to equivalent dose in 2-Gy fractions (EQD2), and the dose became negligible at approximately 9 mm from the cavity edge. A minimum cavity-edge-to-skin distance of 13 mm was therefore prospectively adopted as an additional conservative safety margin. Further details of the dosimetric model, including cavity-wall absorbed doses and radial dose profiles, are provided in Appendix A.

These simulations were intended for protocol design and activity selection and were not designed to provide patient-specific absorbed-dose estimates. Actual absorbed dose may vary according to cavity volume and geometry, local activity distribution, migration or washout, and retention kinetics. Cases with cavity volumes or geometries substantially different from those assumed in the model were considered for additional medical physics review according to institutional procedures.

### 2.8. Post-Injection Imaging and Biodistribution Assessment

Post-injection imaging was performed to assess early localization of [^90^Y]Y-DOTA-biotin within the treated post-VABB target region, document biodistribution, and identify clinically relevant off-target uptake. Whole-body planar bremsstrahlung imaging, including anterior and posterior projections, was acquired using an eight-slice SPECT/CT system (Discovery NM/CT 670, GE Healthcare, Milwaukee, WI, USA) to assess overall biodistribution and identify clinically relevant extra-mammary localization. All 18 treated patients underwent a dedicated ^90Y PET/CT acquisition approximately 1–3 h after administration using a Biograph mCT Flow 20-4R PET/CT system (Siemens Medical Solutions USA, Inc., Hoffman Estates, IL, USA). Imaging findings were assessed primarily qualitatively, with particular attention to early focal localization and clinically relevant off-target uptake. Seven of 18 patients additionally consented to repeated imaging and underwent at least three serial PET/CT acquisitions at different time points for quantitative pharmacokinetic and patient-specific dosimetric analyses. Participation in this additional imaging protocol depended on patient consent and willingness to undergo repeated imaging rather than on predefined clinical selection criteria. Detailed quantitative dosimetric analyses are not included in the present report and will be reported separately.

### 2.9. Surgery and Pathologic Assessment

Breast-conserving surgery was performed 4–7 weeks after intralesional treatment according to routine clinical practice, without protocol-mandated modifications to the surgical procedure or timing.

Surgical specimens were reviewed by two experienced breast pathologists who were blinded to the assigned [^90^Y]Y-DOTA-biotin activity level. All available slides were examined to identify the tumor-bed region and evaluate residual disease.

At gross examination, the tumor-bed region was identified as an area showing loss of normal breast architecture, with fibrous, hemorrhagic, and/or necrotic tissue at the periphery of or within the previous lesion site.

Residual tumor cellularity (RTC) was estimated as the percentage of viable tumor cells within the tumor-bed region, using principles adapted from histopathologic assessment after neoadjuvant chemotherapy. Post-procedural histologic features were evaluated semiquantitatively and expressed as the percentage of the evaluated tumor-bed area showing fibrosis/hyalinization, necrosis, inflammatory infiltrates, and neoangiogenesis. Inflammatory infiltrates included foamy macrophages, lymphocytes, multinucleated giant cells, and hemosiderin-laden macrophages. Morphologically normal breast tissue, when represented in the reviewed sections, was also evaluated descriptively.

No validated radiation-response scoring system or dedicated predefined scoring sheet for radiation-induced histologic changes was used; assessment of post-procedural histologic features was therefore descriptive and exploratory.

For patients with no residual tumor in the surgical specimen, histologic type and grade were assigned on the basis of the diagnostic biopsy material, according to the World Health Organization classification and routine institutional practice. The protocol-specified co-primary pathologic endpoint, breast-only pathologic complete response (pCR), was defined as the absence of residual invasive carcinoma and ductal carcinoma in situ in the resected breast specimen on hematoxylin and eosin examination. Nodal status was not included in this definition because axillary staging was not mandated by the protocol and was not performed uniformly.

### 2.10. Safety Assessments

Safety was assessed from intralesional administration until definitive surgery, which was generally performed 4–7 weeks later, in accordance with the acute safety and feasibility objectives of this phase I trial. Toxicities occurring after surgery were not systematically collected within the study protocol.

Systemic adverse events were graded according to the National Cancer Institute Common Terminology Criteria for Adverse Events, version 5.0. Local injection-site reactions were graded using the Radiation Therapy Oncology Group acute radiation morbidity criteria at protocol-defined visits, including assessments approximately 2 weeks and 4–6 weeks after treatment.

The protocol-defined DLT was any grade ≥ 3 cutaneous adverse event occurring within 10 days after intralesional treatment and considered related to the study treatment or administration procedure by the investigator. The 10-day interval represented the prespecified decision window for cohort progression and activity escalation and was not intended to encompass all potential treatment-related cutaneous toxicity. Safety monitoring therefore continued beyond the DLT window, with local and systemic toxicity assessed at approximately 2 weeks and 4–6 weeks after treatment and safety information collected through definitive surgery. Cutaneous events occurring after Day 10 were recorded and graded as adverse events but were not classified as protocol-defined DLTs for escalation purposes.

Hematologic and standard biochemical tests were obtained at baseline and repeated during follow-up according to the protocol to identify potential systemic effects. Events resulting in treatment discontinuation, hospital admission, or postponement of planned surgery were specifically recorded.

### 2.11. Statistical Analysis

Baseline and treatment characteristics were summarized descriptively for the overall population and by activity level. Continuous variables were summarized using means with ranges or medians with interquartile ranges and ranges, as appropriate. Categorical variables were reported as absolute frequencies and percentages. Given the small cohort size, cohort-level categorical outcomes were presented primarily as absolute numbers with percentages in parentheses. Selected proportions, including the principal safety outcomes and breast-only pathologic complete response, were reported with two-sided 95% confidence intervals calculated using the Wilson score method.

The safety population comprised all participants who received avidin followed by [^90^Y]Y-DOTA-biotin. Safety analyses included all protocol-defined dose-limiting toxicities and treatment-emergent adverse events. Participants who discontinued the study before completing the 10-day dose-limiting toxicity window for reasons unrelated to toxicity were considered non-evaluable for dose-limiting toxicity assessment. All safety information collected before discontinuation was nevertheless included in the descriptive safety analyses. Escalation decisions were based on completion and review of the prespecified acute safety window for each activity cohort.

The protocol-specified co-primary pathologic endpoint, breast-only pCR, was assessed among patients who completed intralesional treatment and underwent definitive surgery. Patients without an evaluable pathologic response assessment were classified as non-responders.

The protocol-specified secondary endpoint was patient-specific absorbed-dose assessment. Serial quantitative imaging and patient-specific dosimetry data were available in a consenting subgroup but were not included in the present statistical analysis and will be reported separately. Additional exploratory analyses in the present report included descriptive evaluation of post-injection biodistribution, the presence or absence of clinically relevant off-target uptake, the feasibility of proceeding to breast-conserving surgery without treatment-related delay, and descriptive post-procedural histologic findings at surgery.

Although preliminary antitumor activity assessed as breast-only pCR was a protocol-specified co-primary objective, the completed activity-escalation phase was not powered for formal efficacy testing or comparisons among activity levels. Accordingly, the co-primary pathologic endpoint and the secondary and exploratory outcomes included in the present report were analyzed descriptively. No formal hypothesis testing or type I error control was planned. Missing safety data were not imputed. Statistical analyses were performed using SAS software, version 9.4 (SAS Institute Inc., Cary, NC, USA).

## 3. Results

### 3.1. Patient and Lesion Characteristics

Between July 2024 and January 2025, 18 women were enrolled and received the assigned intralesional treatment. All participants completed the prespecified 10-day dose-limiting toxicity observation period and subsequently underwent breast-conserving surgery.

The median age was 57 years (range, 48–74 years). All patients had nonpalpable breast lesions meeting the eligibility criteria, including a maximum lesion diameter of ≤15 mm and a minimum skin-to-cavity distance of ≥13 mm. The median lesion size at presentation was 10 mm (interquartile range [IQR], 6–12 mm; range, 5–14 mm).

Histologic examination of the diagnostic VABB specimens identified pure ductal carcinoma in situ (DCIS) in 5 patients, invasive carcinoma of no special type (NST), including one case with associated DCIS, in 7 patients, invasive lobular carcinoma (ILC) in 4 patients, mixed ILC/NST carcinoma in 1 patient, and tubular carcinoma in 1 patient.

Most invasive tumors were histologic grade 1 or 2; one patient had a grade 3 pleomorphic invasive lobular carcinoma. Among evaluable invasive carcinomas, estrogen receptor expression ranged from approximately 50% to 95%, progesterone receptor expression from 40% to 95%, and Ki-67 was generally low (2–15%), except for a value of 35% in the pleomorphic invasive lobular carcinoma. HER2 status was available in 12 of 13 invasive carcinomas: 5 had an immunohistochemical score of 0 and 7 had a score of 1+. Given the single-arm phase I design and the limited sample size, baseline characteristics were analyzed descriptively, and no comparative inferences were planned.

### 3.2. Treatment Delivery and Subsequent Surgery

All 18 patients received same-session sequential intralesional administration of avidin followed immediately by [^90^Y]Y-DOTA-biotin at the assigned activity level.

Three sequential cohorts of six patients each were treated. At activity level A, corresponding to a nominal target activity of 28 MBq, the mean administered activity was 34 MBq (range, 27–37 MBq). At activity level B, corresponding to a nominal target activity of 57 MBq, the mean administered activity was 62 MBq (range, 58–65 MBq). At activity level C, corresponding to a nominal target activity of 126 MBq, the mean administered activity was 125 MBq (range, 118–134 MBq). Differences between nominal target and measured administered activities reflected technical variability related to radiopharmaceutical preparation, dispensing, activity measurement, and timing of administration and did not alter protocol-defined cohort assignment.

All patients underwent breast-conserving surgery 4–7 weeks after intralesional treatment. No treatment-related postponements, modifications of the planned surgical procedure, or protocol deviations affecting surgery were observed.

### 3.3. Post-Injection Imaging and Biodistribution

**Figure 2:**
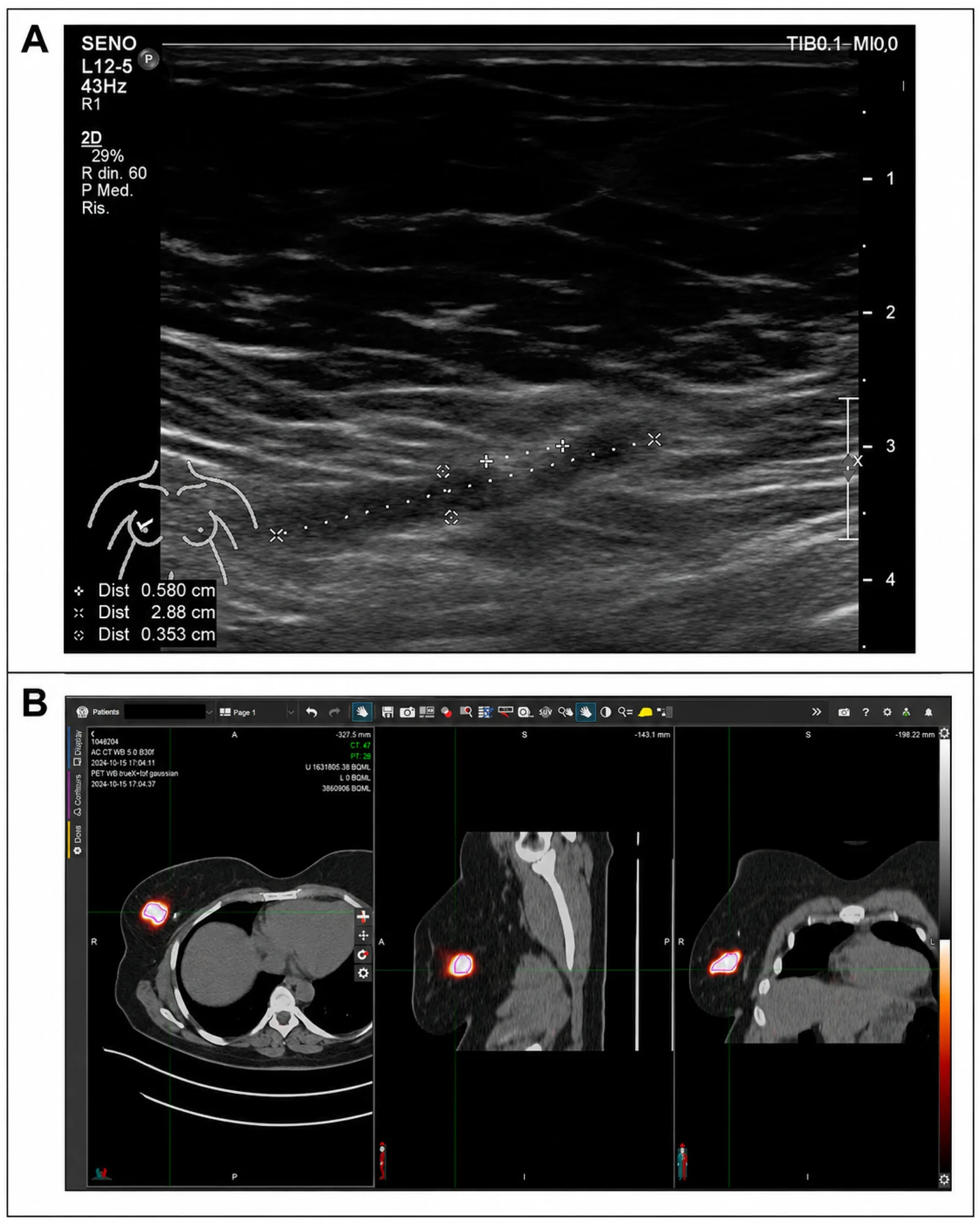
Ultrasound-guided treatment administration and early focal localization on ^90Y PET/CT. (**A**) Ultrasound image showing the post-VABB target region during ultrasound-guided intralesional treatment. (**B**) Dedicated breast ^90Y PET/CT acquired approximately 1 h after administration, demonstrating early focal activity centered within the treated post-VABB target region, without clinically relevant off-target uptake on qualitative assessment. In the PET/CT panel, the color scale represents relative ^90Y activity intensity, with warmer colors indicating higher activity; crosshairs indicate the focal activity localization. Ultrasound calipers and symbols represent standard imaging-system measurement annotations.

All 18 treated patients underwent dedicated breast ^90Y PET/CT approximately 1–3 h after administration. Qualitative assessment demonstrated early focal localization of activity centered within the treated post-VABB target region, without clinically relevant extra-lesional uptake at the time of imaging (Figure 2). Whole-body planar bremsstrahlung imaging showed no clinically relevant extra-mammary localization. Representative images from the three nominal activity levels are shown in Supplementary Figure S1. Seven of 18 patients (38.9%) additionally underwent at least three serial PET/CT acquisitions at different time points for quantitative pharmacokinetic and patient-specific dosimetric analyses. Participation in the serial-imaging subgroup was based on additional consent and willingness to undergo repeated imaging and was not determined by predefined clinical selection criteria. Detailed quantitative pharmacokinetic and patient-specific dosimetric analyses are not included in the present report and will be reported separately.

### 3.4. Safety

No protocol-defined DLT was observed among the 18 DLT-evaluable patients (0/18; 0%; two-sided 95% Wilson CI, 0.0–17.6%), and no grade ≥ 3 adverse event occurred (0/18; 0%; two-sided 95% Wilson CI, 0.0–17.6%). No DLT occurred in any of the three six-patient activity cohorts (0/6 at each level; two-sided 95% Wilson CI, 0.0–39.0%).

All recorded treatment-emergent adverse events were grade 1. Injection-site pain was reported in 4 of 18 patients (22.2%), and erythema or skin redness was reported in 3 patients (16.7%).

Pain occurred in 1 of 6 patients at activity level A, 1 of 6 patients at activity level B, and 2 of 6 patients at activity level C. Pain was self-limited and lasted 1 day at activity level A, 8 days at activity level B, and 1–2 days at activity level C. Erythema or skin redness occurred in 2 of 6 patients at activity level B and 1 of 6 patients at activity level C and resolved within approximately 1 day in all cases.

Other grade 1 treatment-emergent events included hematoma in 4 patients (22.2%), as well as isolated cases of neutropenia, leukopenia, lymphopenia, pruritus, burning sensation, increased alanine aminotransferase, and increased direct bilirubin. No clinically relevant hematologic toxicity, treatment discontinuation, hospitalization, or postponement or modification of definitive breast-conserving surgery occurred. No clear descriptive trend toward an increase in the frequency or severity of adverse events was observed across the three activity levels.

Treatment-emergent adverse events according to nominal [^90^Y]Y-DOTA-biotin activity level are summarized in Table 1.

### 3.5. Pathologic Outcome

For the protocol-specified co-primary objective of preliminary antitumor activity, breast-only pCR, defined as the absence of residual tumor cellularity (RTC) from invasive or in situ carcinoma in the breast surgical specimen, was observed in 5 of 18 patients, corresponding to a rate of 27.8% (95% confidence interval [CI], 12.5–50.9%). In this report, this finding should be interpreted descriptively as the absence of residual carcinoma at surgery rather than as evidence of a treatment-induced response.

By activity level, breast-only absence of RTC at surgery was observed in 1/6 patients in cohort A (16.7%; 95% CI, 3.0–56.4%), 2/6 in cohort B (33.3%; 95% CI, 9.7–70.0%), and 2/6 in cohort C (33.3%; 95% CI, 9.7–70.0%). Because of the small cohort sizes, single-arm design, and descriptive nature of the analysis, no formal comparisons among activity levels were performed, and no relationship between administered activity and pathologic response can be inferred. Importantly, VABB was performed as a diagnostic procedure, and the proportion of the original malignant lesion removed during VABB was not prospectively quantified on a per-patient basis. Consequently, residual tumor burden immediately before ARTHE treatment cannot be reliably reconstructed. The observed absence of residual carcinoma at surgery therefore cannot be attributed specifically to the radionuclide treatment or distinguished from the tissue-removing effect of VABB, and no treatment-specific efficacy conclusions can be drawn from this phase I study.

### 3.6. Histologic Features of the Tumor Bed

In the surgical specimens, the tumor-bed region, encompassing residual carcinoma when present together with post-procedural tissue changes, measured 6–14 mm in greatest extent. Among patients with residual carcinoma, estimated residual tumor cellularity (RTC) varied substantially, ranging from approximately 5% to 80%.

Fibrosis or hyalinization was consistently identified and involved approximately 20–80% of the evaluated tumor-bed area. Neoangiogenesis was also observed, involving approximately 3–15% of the tumor-bed area.

Inflammatory infiltrates represented a prominent histologic component and involved approximately 5–73% of the tumor-bed area. These comprised mixed populations of lymphocytes, granulocytes, foamy and/or hemosiderin-laden macrophages, and multinucleated giant cells.

Necrosis was absent in all but one case. In one patient with microinvasive carcinoma, a minimal necrotic component was identified, involving approximately 3% of the tumor-bed area in the representative section.

These findings were interpreted as post-procedural histologic changes. On routine histologic examination, changes related to mechanical tissue injury and repair following VABB could not be reliably distinguished from changes potentially associated with the study treatment.

**Figure 3:**
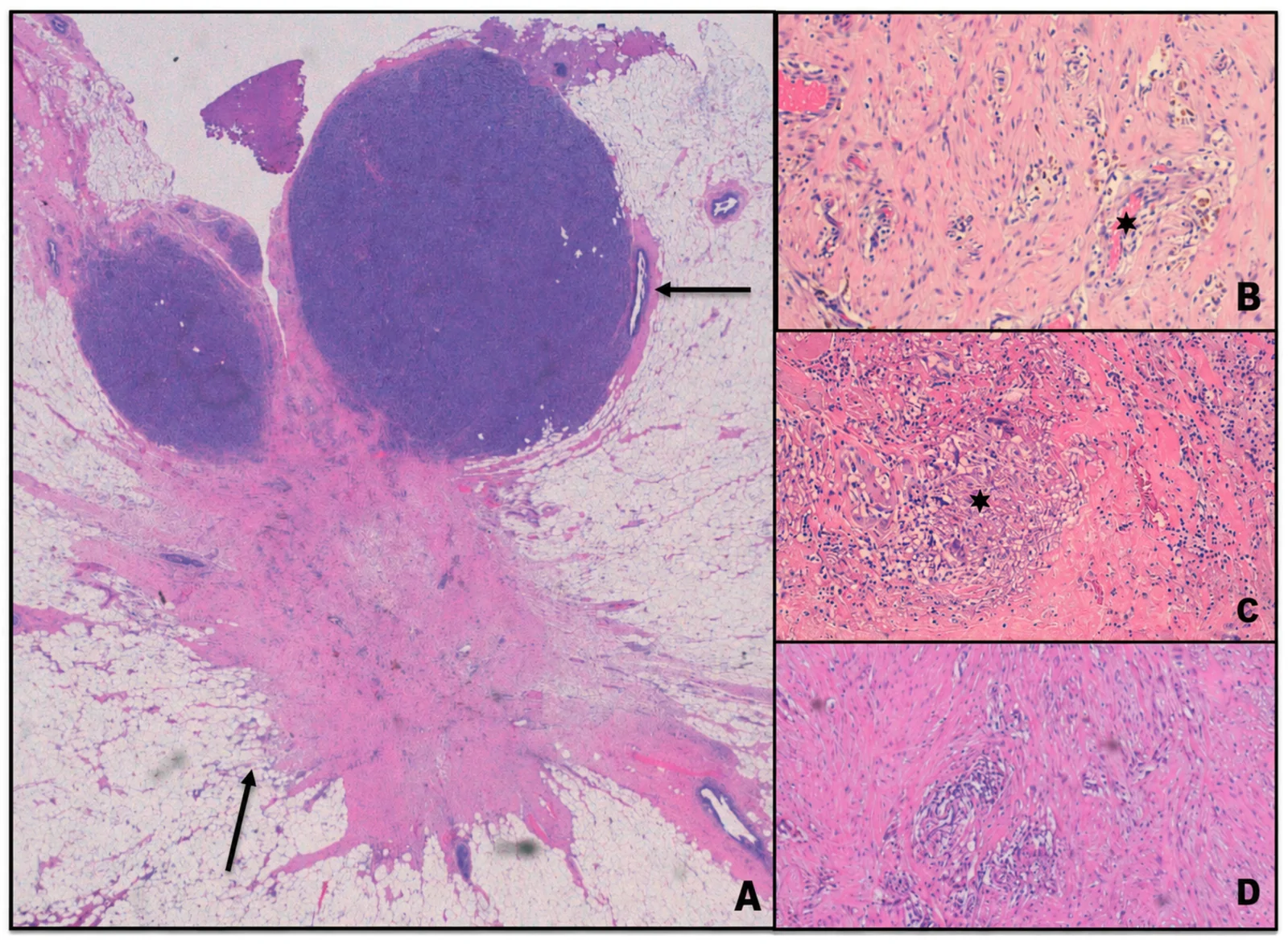
Representative histopathologic features of the post-VABB tumor-bed region after intralesional avidin-mediated [^90^Y]Y-DOTA-biotin administration. (**A**) Low-power whole-section view of a representative surgical specimen showing residual carcinoma (horizontal arrow) and fibrotic tumor-bed tissue without residual tumor cellularity (RTC) (vertical arrow). (**B**–**D**) Higher-magnification hematoxylin-and-eosin-stained views of tumor-bed areas without RTC, showing fibrosis with neoangiogenesis ((**B**), asterisks), focal necrosis ((**C**), asterisk), and fibrosis with inflammatory infiltrates (**D**). Original magnification: ×20 for panels (**B**–**D**).

Representative histopathologic features are shown in Figure 3.

**Figure 4:**
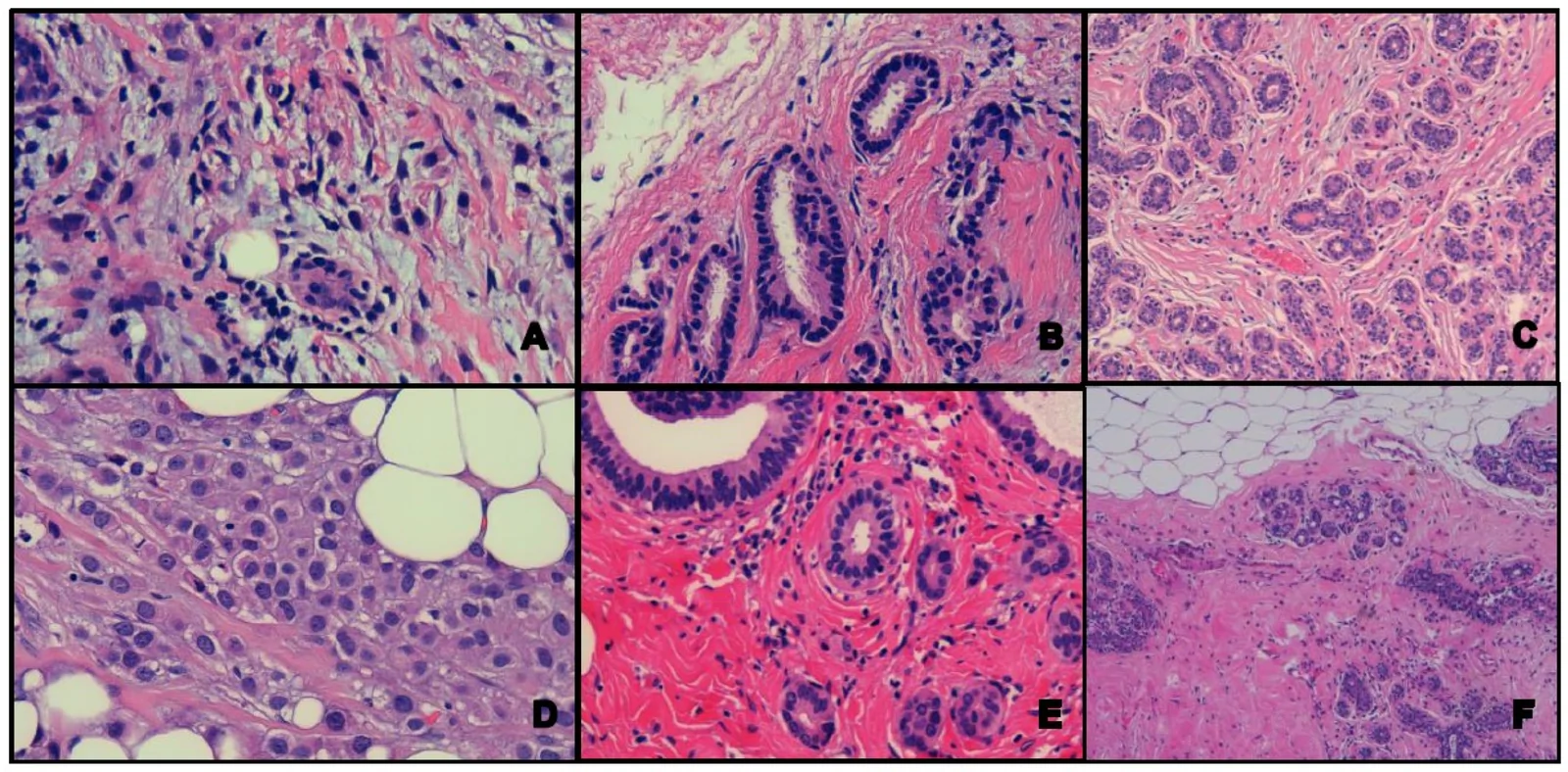
Representative cytologic morphology in diagnostic VABB and surgical specimens. Representative hematoxylin-and-eosin-stained sections illustrating invasive lobular carcinoma (ILC; (**A**,**D**)), invasive carcinoma of no special type (NST; (**B**,**E**)), and morphologically normal breast tissue (**C**,**F**) in diagnostic VABB (**A**–**C**) and surgical specimens (**D**–**F**). No overt treatment-specific nuclear abnormality was apparent in the representative surgical sections shown. Original magnification: ×40 for panels (**A**,**B**,**D**,**E**); ×10 for panels (**C**,**F**).

In the morphologically normal breast parenchyma represented in the reviewed surgical sections, no overt treatment-specific morphologic abnormality was identified. Representative comparisons of nuclear morphology in diagnostic VABB and surgical specimens are shown in Figure 4. However, normal-tissue radiation injury was not assessed using a prespecified histologic scoring system or systematic spatial sampling.

## 4. Discussion

In this investigator-initiated, single-center phase I activity-escalation trial, same-session sequential intralesional administration of avidin followed by [^90^Y]Y-DOTA-biotin into the post-VABB target region was feasible, well tolerated in the acute and short-term setting, and compatible with standard breast-conserving surgery. No protocol-defined dose-limiting toxicities, grade ≥ 3 adverse events, clinically relevant hematologic toxicity, treatment discontinuations, hospitalizations, or treatment-related surgical delays were observed across the three activity levels. Qualitative post-injection imaging consistently demonstrated early focal localization of the radiopharmaceutical within the treated post-VABB target region, without clinically relevant extra-lesional uptake. These findings provide early clinical support for the feasibility of integrating a localized radionuclide strategy into the interval between diagnostic biopsy and definitive surgery in selected patients with nonpalpable breast cancer.

The clinical rationale for this approach is based on the post-biopsy setting. VABB is an established minimally invasive diagnostic procedure for nonpalpable breast lesions because of its high diagnostic accuracy, large tissue yield, and favorable safety profile [1,2]. In selected benign lesions, it may also achieve complete lesion removal [3,4]. In malignant disease, however, VABB is not considered definitive treatment, even when post-biopsy imaging suggests complete removal of the target lesion or associated microcalcifications [5,6,7,8,9,10]. Microscopic residual disease may remain within or around the biopsy cavity, and definitive surgery therefore continues to represent the standard of care. At the same time, the interval between VABB and surgery creates a temporary opportunity in which the post-VABB target region remains directly accessible for localized treatment. ARTHE was designed to exploit this opportunity without replacing or modifying standard surgical management. Its immediate objective was to determine whether avidin and [^90^Y]Y-DOTA-biotin could be administered sequentially into the post-VABB target region, demonstrate early focal localization on post-injection imaging, and allow subsequent breast-conserving surgery without delay or added acute morbidity. ARTHE differs substantially in treatment configuration and procedure from the historical IART approach, in which avidin was administered intraoperatively to the surgical bed and radiolabeled biotin was subsequently administered intravenously after a 12–24 h interval. In ARTHE, both components are instead administered sequentially by the intralesional route during the same treatment session. The strategy is therefore more appropriately interpreted as avidin-mediated local trapping rather than conventional systemic pretargeting. This same-session approach avoids intravenous administration of the radiolabeled ligand and eliminates the interval between administration of the two components, while allowing the investigational treatment to be completed entirely between diagnostic VABB and definitive surgery. These differences are procedural and practical; the present study was not designed as a comparative study and therefore cannot establish superiority over IART in terms of safety, dosimetry, radiopharmaceutical localization, or efficacy.

The favorable acute safety profile was observed across all three activity cohorts, with no clear descriptive trend toward increased frequency or severity of adverse events at higher activity levels. Local treatment-emergent events were grade 1 and included pain, erythema or skin redness, pruritus, burning sensation, and hematoma, and no clinically relevant systemic toxicity was identified. Importantly, all patients proceeded to breast-conserving surgery within the planned 4–7-week interval. In the morphologically normal breast parenchyma represented in the reviewed surgical sections, no overt treatment-specific morphologic abnormality was identified. However, normal-tissue radiation injury was not assessed using a prespecified histologic scoring system or systematic spatial sampling. Together with qualitative imaging showing early focal localization without clinically relevant extra-lesional uptake, these observations support the feasibility of the treatment approach but do not establish the absence of normal-tissue injury or long-term tissue effects. The primarily qualitative imaging assessment and the absence of patient-specific dosimetric results in the present report preclude conclusions regarding individual absorbed dose, while the limited duration of safety follow-up precludes assessment of delayed toxicity.

Breast-only absence of residual tumor cellularity (RTC), corresponding to the protocol-defined breast-only pCR endpoint, was observed in 5 of 18 patients (27.8%). Although preliminary antitumor activity assessed by this pathologic endpoint was a protocol-specified co-primary objective, the finding should be interpreted descriptively as the absence of residual carcinoma at surgery rather than as evidence of a treatment-induced response. The activity-escalation phase was neither powered to establish treatment efficacy nor designed with a post-VABB control group. Moreover, VABB was performed as a diagnostic procedure, and the proportion of the original malignant lesion removed during biopsy was not prospectively quantified on a per-patient basis. Therefore, residual tumor burden immediately before ARTHE cannot be reliably reconstructed, and the respective contributions of mechanical tissue removal and radionuclide treatment cannot be distinguished. Comparison of pre-VABB lesion dimensions with post-biopsy cavity size would not resolve this limitation because cavity size is not a surrogate for residual malignant burden. The observed 27.8% rate should therefore be regarded as a descriptive, hypothesis-generating finding rather than as evidence of treatment efficacy. A controlled study including an appropriate post-VABB comparator will be required to determine the specific therapeutic contribution of ARTHE.

Histologic examination of the post-VABB tumor-bed region showed a heterogeneous spectrum of stromal and inflammatory changes, including fibrosis/hyalinization, inflammatory infiltrates, hemosiderin-laden macrophages, multinucleated giant cells, and neoangiogenesis. These findings should be interpreted cautiously, as they are not specific to radionuclide exposure and may also represent the expected tissue-repair response following VABB. In the absence of an untreated post-VABB control group, the respective contributions of biopsy-related tissue injury and radionuclide exposure cannot be distinguished. Accordingly, these findings are described as post-procedural tissue changes rather than treatment-induced or radiation-induced effects. No individual histologic feature was considered specific to the radionuclide treatment, and the semiquantitative observations should therefore be regarded as descriptive and exploratory rather than as evidence of a treatment-specific histologic response.

The biological and technological rationale for ARTHE is supported by prior clinical and translational experience with avidin- or streptavidin–biotin platforms followed by radiolabeled biotin administration. In breast cancer, the IART program demonstrated that locally administered avidin can persist at the injection site and support subsequent localization of radiolabeled biotin administered either intravenously or locally [11,12,13,14]. ARTHE used native avidin supplied as an investigational medicinal product by Diatheva S.r.l. and the DOTA-conjugated biotin derivative r-BHD labeled with ^90Y and prepared at the IRST Radiopharmacy. Although these products are based on the same avidin–biotin targeting principle used in IART, pharmaceutical identity with the preparations employed in the historical IART studies cannot be established.

Related pretargeted radionuclide strategies have also shown clinical feasibility in high-grade glioma, non-Hodgkin lymphoma, and metastatic colorectal cancer [17,18,19,20,21,22,23,24]. Collectively, these studies support the translational potential of avidin/streptavidin–biotin targeting, while also highlighting limitations related to immunogenicity, procedural complexity, and the importance of selecting an appropriate clinical setting. ARTHE extends this concept to a setting in which the target is locally accessible, anatomically defined, and temporarily available after biopsy.

The proposed mechanism relies on both the exceptionally high affinity of avidin for biotin and the local characteristics of the post-VABB tissue environment. Avidin is a cationic glycoprotein, and previous experimental and clinical observations suggest that inflammatory or biologically altered tissues may favor local retention of avidin-based constructs [11,24,25,26]. The inflammatory and reparative microenvironment induced by VABB may therefore contribute to persistence of intralesionally administered avidin and facilitate subsequent trapping of [^90^Y]Y-DOTA-biotin. The early focal localization observed on post-injection imaging is consistent with this hypothesis but does not, by itself, establish the underlying retention mechanism. Because ARTHE did not include a [^90^Y]Y-DOTA-biotin-only comparator, the specific contribution of avidin-mediated local trapping relative to simple intralesional depot administration cannot be determined from the present study.

The three nominal activity levels were prospectively derived from Monte Carlo dosimetric modelling, whereas progression between activity cohorts was governed by the protocol-defined safety and DLT rules. Using a simplified spherical 1 mL cavity model and a planning absorbed dose of 200 Gy to potential microscopic residual disease, activities of 28, 57, and 126 MBq were calculated to extend the 200 Gy isodose to 1, 2, and 3 mm beyond the cavity edge, respectively. The 200-Gy value represented a pragmatic protocol-planning assumption informed by previous radionuclide-therapy experience and should not be interpreted as a validated tumoricidal dose threshold for breast cancer. Accordingly, 126 MBq represented the highest prespecified model-derived activity investigated in this study and should not be interpreted as a maximum tolerated or therapeutically optimal activity. Detailed information on the dosimetric model and activity prescription is provided in Appendix A.

These calculations were intended for protocol planning and cannot substitute for patient-specific dosimetry. Actual absorbed dose may differ substantially among patients because of variations in cavity volume and geometry, local activity distribution, washout or migration, and retention kinetics. Consequently, administered activity cannot be regarded as a direct surrogate for patient-specific absorbed dose, and the present study cannot establish dose–toxicity, dose–histology, or dose–response relationships. Patient-specific quantitative dosimetry obtained in the consenting serial-imaging subgroup will be reported separately. If its therapeutic contribution is confirmed in controlled studies, this approach could be integrated after diagnostic VABB as part of a minimally invasive strategy directed at microscopic residual disease. Relevant prospective endpoints would include residual tumor burden, surgical margin status, re-excision rate, acute and late toxicity, cosmetic outcomes, patient-reported outcomes, local control, and effects on the diagnostic-to-surgical workflow. Any future consideration of surgical de-escalation or omission would require substantially stronger evidence, carefully selected populations, reliable imaging and pathologic response criteria, multidisciplinary oversight, and long-term oncologic follow-up. The present findings do not support any modification of standard surgical management.

The study has several strengths. It was prospective and investigator-initiated, used predefined activity levels and safety rules, applied standardized eligibility criteria including a conservative skin-distance constraint, and incorporated post-injection imaging to assess radiopharmaceutical localization. It was conducted in an academic, certified phase I nuclear medicine unit with dedicated radiopharmacy, medical physics, radiation-protection, imaging, and clinical-trial infrastructure. Several limitations should nevertheless be acknowledged. The study was conducted at a single center and included only 18 patients. It had no control group and included a heterogeneous population with respect to histologic subtype, consistent with the phase I setting. Although preliminary antitumor activity assessed as breast-only pCR was a protocol-specified co-primary objective, the activity-escalation phase was not powered to establish treatment efficacy or to compare activity levels. The inclusion of both in situ and invasive carcinomas and of multiple histologic subtypes further limits interpretation of the pathologic findings. Imaging assessment was primarily qualitative, whereas quantitative imaging and patient-specific dosimetry were available only in a subgroup and were not included in the present report.

The 10-day DLT window was designed specifically for activity-escalation decisions and should not be interpreted as encompassing the full temporal spectrum of radiation-related toxicity. Although local and systemic safety assessments continued through definitive surgery at 4–7 weeks, systematic long-term safety follow-up and dedicated cosmetic outcome assessment were not prospectively planned. Therefore, delayed effects such as fibrosis, fat necrosis, breast atrophy, telangiectasia, chronic pain, and cosmetic changes cannot be assessed from the present data. The eligibility criteria also limit generalizability. This first-in-human study was restricted to patients with small (≤15 mm), clinically nonpalpable lesions and a minimum skin-to-cavity distance of 13 mm. Because a denominator-based screening population was not prospectively collected, the proportion of patients with nonpalpable breast cancer who would meet all eligibility criteria cannot be reliably estimated. Moreover, the present safety and feasibility findings cannot be directly extended to larger or palpable tumors, more superficial lesions, or different post-biopsy cavity geometries, which would require dedicated dosimetric and safety evaluation.

A further major limitation concerns interpretation of the pathologic findings. Because diagnostic VABB may remove part or all of the original malignant lesion and the extent of lesion removal was not prospectively quantified on a per-patient basis, breast-only absence of residual tumor cellularity (RTC) at surgery cannot be attributed specifically to the radionuclide treatment. The respective contributions of mechanical tissue removal and treatment effect therefore cannot be distinguished in this single-arm study. Moreover, the stromal and inflammatory changes observed in the tumor-bed region, including fibrosis/hyalinization, inflammatory infiltrates, and multinucleated giant-cell reactions, are nonspecific. Because VABB itself induces tissue injury and repair and the study did not include an untreated post-VABB control group, biopsy-related changes cannot be distinguished from possible effects of radionuclide exposure.

## 5. Conclusions

This first-in-human phase I study supports the feasibility of post-VABB intralesional avidin-mediated delivery of [^90^Y]Y-DOTA-biotin, with favorable acute and short-term tolerability, early focal localization on post-injection imaging, and compatibility with standard breast-conserving surgery. Controlled studies incorporating quantitative imaging and patient-specific dosimetry are warranted to determine the specific therapeutic contribution of this post-VABB target-region radionuclide approach.

## Figures and Tables

**Table 1:** Treatment-emergent adverse events according to nominal [^90^Y]Y-DOTA-biotin activity level Data are presented as *n* (%). Cohort headings indicate the prespecified nominal target activity levels. All listed treatment-emergent adverse events were grade 1. No grade 2 events, protocol-defined dose-limiting toxicities, or grade ≥ 3 adverse events were observed.

Adverse Event	Total (*n* = 18), *n* (%)	28 MBq (*n* = 6), *n* (%)	57 MBq (*n* = 6), *n* (%)	126 MBq (*n* = 6), *n* (%)
Pain	4 (22.2)	1 (16.7)	1 (16.7)	2 (33.3)
Erythema/skin redness	3 (16.7)	0 (0.0)	2 (33.3)	1 (16.7)
Hematoma	4 (22.2)	3 (50.0)	0 (0.0)	1 (16.7)
Neutropenia	1 (5.6)	0 (0.0)	1 (16.7)	0 (0.0)
Leukopenia	1 (5.6)	0 (0.0)	1 (16.7)	0 (0.0)
Pruritus	1 (5.6)	1 (16.7)	0 (0.0)	0 (0.0)
ALT increase	1 (5.6)	0 (0.0)	1 (16.7)	0 (0.0)
Direct bilirubin increase	1 (5.6)	0 (0.0)	1 (16.7)	0 (0.0)
Burning sensation	1 (5.6)	0 (0.0)	1 (16.7)	0 (0.0)
Lymphopenia	1 (5.6)	0 (0.0)	0 (0.0)	1 (16.7)

Abbreviations: ALT, alanine aminotransferase.

**Figure A1:**
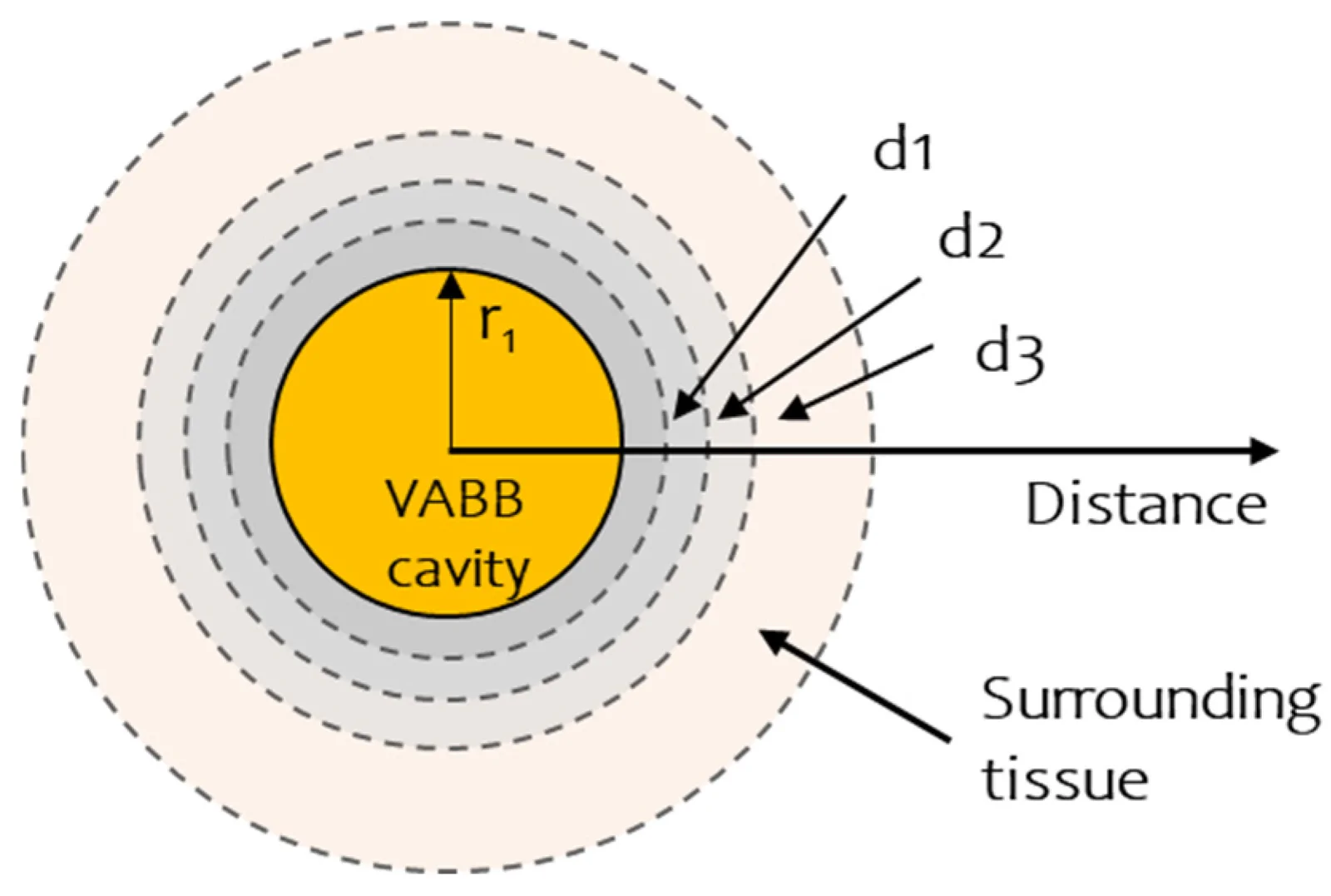
Irradiation geometry: the yellow sphere represents the cavity uniformly filled with Y-90; the three shells surrounding the cavity are 0.1, 0.2 and 0.3 cm thick, respectively. d1, d2 and d3 represent the distances from the cavity edge at which the 200 Gy dose is prescribed.

**Figure A2:**
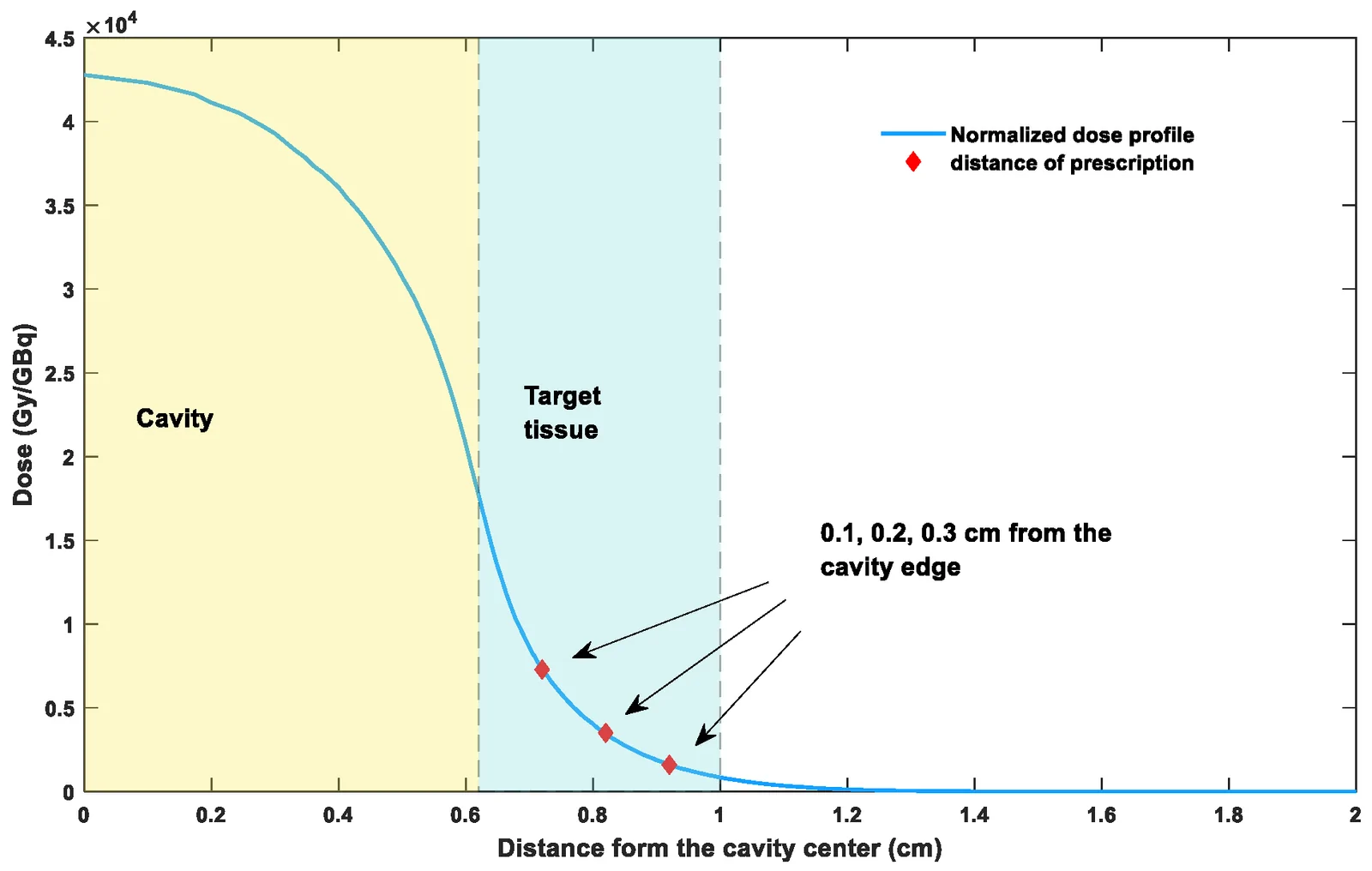
Normalized dose profile as a function of the distance from the cavity center. The yellow region corresponds to the cavity while the light blue region is the target tissue. The red markers are the distances at which the 200 Gy dose is prescribed.

**Figure A3:**
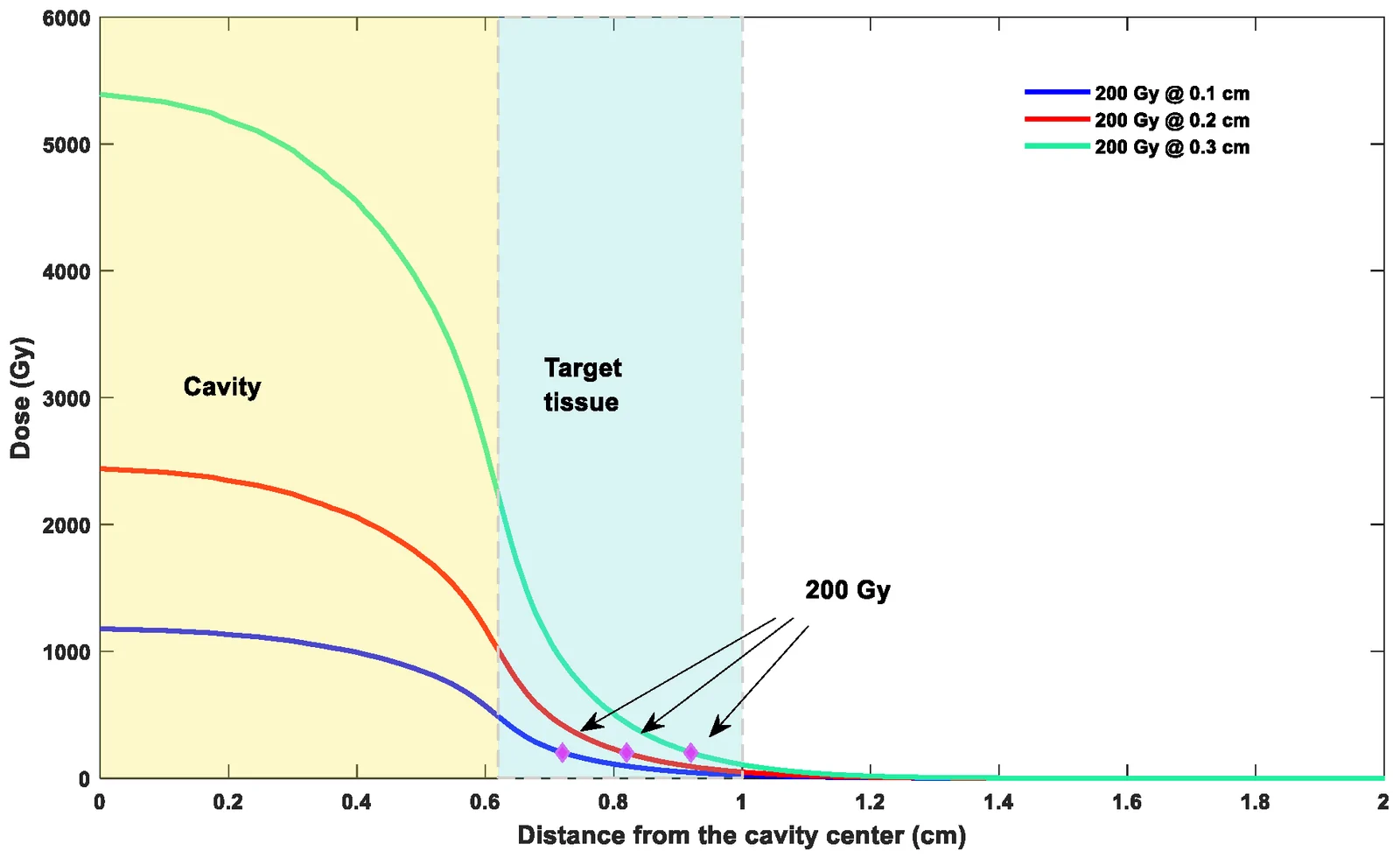
Absorbed dose profile as a function of the distance from the cavity center. The yellow region corresponds to the cavity while the light blue region is the target tissue. The magenta markers are the distances at which the 200 Gy dose is prescribed.

**Figure A4:**
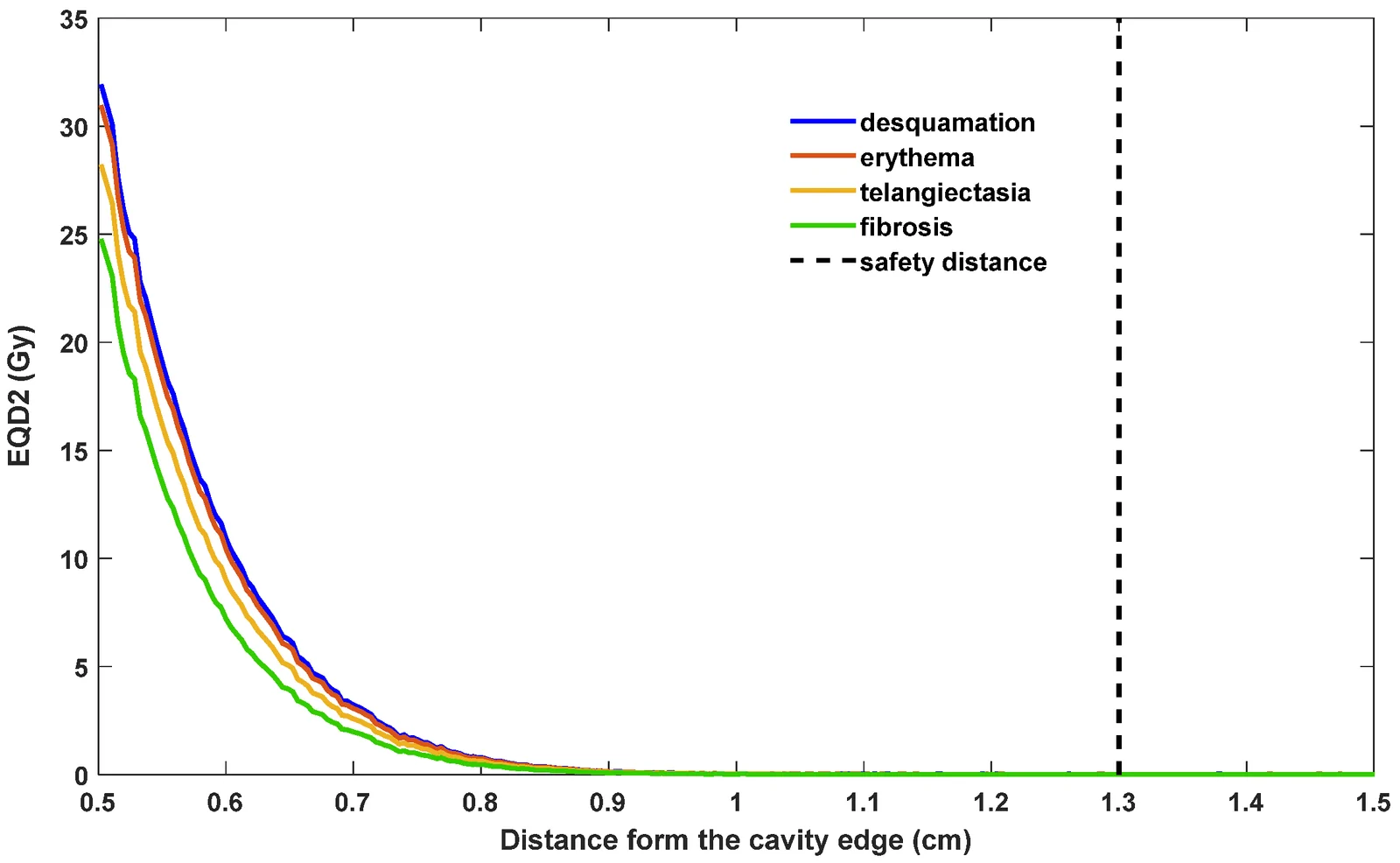
EQD2 as a function of the distance from the cavity edge for desquamation (blue line), erythema (red line), telangiectasia (orange line) and fibrosis (green line). The vertical line represents the minimum distance between the cavity edge and the skin layer adopted as a conservative safety margin. The x-axis starts at 0.5 cm.

## Data Availability

The data presented in this study are not publicly available because of patient privacy, ethical, and regulatory restrictions. Deidentified individual-level data and supporting methodological documents may be made available by the corresponding author upon reasonable request, subject to approval by the relevant institutional review bodies and applicable data-protection regulations.

## Supplementary Materials

The following supporting information can be downloaded at: https://www.mdpi.com/article/10.3390/cancers18172760/s1, Figure S1: Representative whole-body bremsstrahlung images after intralesional [^90^Y]Y-DOTA-biotin administration.

## Abbreviations

AIFA	Italian Medicines Agency
ALT	Alanine aminotransferase
ARTHE	Avidination for Radionuclide Therapy in Nonpalpable Breast Cancer
BI-RADS	Breast Imaging Reporting and Data System
CI	Confidence interval
CTCAE	Common Terminology Criteria for Adverse Events
DLT	Dose-limiting toxicity
DOTA	1,4,7,10-tetraazacyclododecane-1,4,7,10-tetraacetic acid
ECOG	Eastern Cooperative Oncology Group
GCP	Good Clinical Practice
GMP	Good Manufacturing Practice
HER2	Human epidermal growth factor receptor 2
HPLC	High-performance liquid chromatography
IART	Intraoperative avidination for radionuclide therapy
IQR	Interquartile range
IRST	Istituto Romagnolo per lo Studio dei Tumori
PET/CT	Positron emission tomography/computed tomography
pCR	Pathologic complete response
r-BHD	Reduced biotinamidohexylamine-DOTA
RTC	Residual tumor cellularity
RTOG	Radiation Therapy Oncology Group
SPECT/CT	Single-photon emission computed tomography/computed tomography
TLC	Thin-layer chromatography
VABB	Vacuum-assisted breast biopsy
WHO	World Health Organization

## Appendix A. Dosimetric Modelling and Activity Prescription

The dosimetric planning objective was to deliver an absorbed dose of 200 Gy to potential microscopic residual disease in the tissue surrounding the post-VABB cavity. The 200-Gy value was adopted as a pragmatic protocol-planning assumption and should not be interpreted as a validated tumoricidal dose threshold for breast cancer. The volume and geometry of the post-VABB region may vary according to the amount of tissue removed and the resulting local anatomy. For dosimetric modelling purposes, an idealized spherical cavity with a volume of 1 mL was assumed. The cavity was modelled as being uniformly filled with ^90Y activity and was considered the source region, whereas the surrounding tissue was considered the target region (Figure A1). The source geometry was simulated using the PENELOPE Monte Carlo code [27,28,29], and the normalized absorbed-dose profile (Gy/GBq) was calculated as a function of distance from the centre of the sphere.

A 10% washout from the modeled source region was assumed for treatment-planning calculations, corresponding to an assumed 90% local retention of activity. This was a model assumption and was not derived from patient-specific retention measurements.

For the assumed 1 mL spherical geometry, the activity required to deliver 200 Gy at different distances from the cavity edge was calculated. Three target distances were considered: 0.1, 0.2, and 0.3 cm, corresponding to d1, d2, and d3, respectively (Figure A1). The radius of a 1 mL spherical cavity is approximately 0.62 cm; therefore, these target distances correspond to radial distances of approximately 0.72, 0.82, and 0.92 cm from the centre of the cavity.

The normalized dose profile obtained from the PENELOPE simulation was used to calculate the activity required to achieve 200 Gy at each of the three target distances. The resulting activity levels were 28, 57, and 126 MBq, respectively (Table A1).

**Table A1:** Activity levels required to deliver the prescribed planning dose of 200 Gy at the specified distances from the cavity edge, together with the corresponding absorbed dose at the cavity wall.

Activity (MBq)	Distance from Cavity Edge (cm)	Prescribed Dose (Gy)	Dose at Cavity Wall (Gy)
28	0.1	200	501.4
57	0.2	200	1039.2
126	0.3	200	2297.2

Figure A2 shows the normalized absorbed-dose profile as a function of distance from the centre of the cavity for the simulated geometry. The yellow region represents the cavity, whereas the light-blue region represents the surrounding target tissue. The positions corresponding to d1 = 0.1 cm, d2 = 0.2 cm, and d3 = 0.3 cm from the cavity edge are indicated.

Figure A3 shows the absorbed-dose profiles in the cavity and surrounding tissue for the three activity levels derived from the Monte Carlo simulations.

The activity levels should therefore be interpreted as dosimetrically derived treatment levels corresponding to different assumed target distances from the modeled cavity, rather than as empirically determined or toxicity-derived activity levels. In particular, 126 MBq represents the highest prespecified model-derived activity investigated in the present study and should not be interpreted as a maximum tolerated or therapeutically optimal activity.

### Radiobiological Modelling of Potential Skin Effects

Intralesional administration of [^90^Y]Y-DOTA-biotin may result in irradiation of the overlying skin. A radiobiological evaluation was therefore performed during treatment planning to define a conservative minimum distance between the modeled source region and the skin and thereby minimize the potential risk of radiation-induced skin effects.

The absorbed-dose profiles were analyzed as a function of distance from the cavity edge. Because available reference data for skin tolerance are largely derived from external beam radiotherapy (EBRT) [30,31], absorbed doses were converted to equivalent dose in 2-Gy fractions (EQD2) according to the formalism described in [32]. The radiobiological parameters used for the EQD2 calculations were derived from references [30,31,32]. To provide a conservative assessment, calculations were performed for the scenario in which 200 Gy was prescribed at 0.3 cm from the cavity edge, corresponding to the highest investigated activity level (126 MBq); the lower activity scenarios yielded lower estimated skin doses.

Figure A4 shows the calculated EQD2 profiles for fibrosis, telangiectasia, erythema, and desquamation. For all skin effects considered, EQD2 decreased rapidly with increasing distance from the cavity edge, from approximately 30 Gy at 0.5 cm to values approaching zero at approximately 0.9 cm. On the basis of this model, a minimum skin-to-cavity distance of 1.3 cm was adopted prospectively as a conservative eligibility constraint.
