# Supplementary material for: Post-VABB Cavity-Targeted Avidin-Pretargeted [90Y]Y-DOTA-Biotin in Nonpalpable Breast Cancer: A Phase I Activity-Escalation Study (ARTHE)
Source: Cancers (Basel). 2026 Aug 25;18(17):2760. doi: 10.3390/cancers18172760 (PMC13564483; doi:10.3390/cancers18172760)
Supplement: Supplementary file 1 [file cancers-18-02760-s001.zip › cancers-4494967-supplementary.pdf]

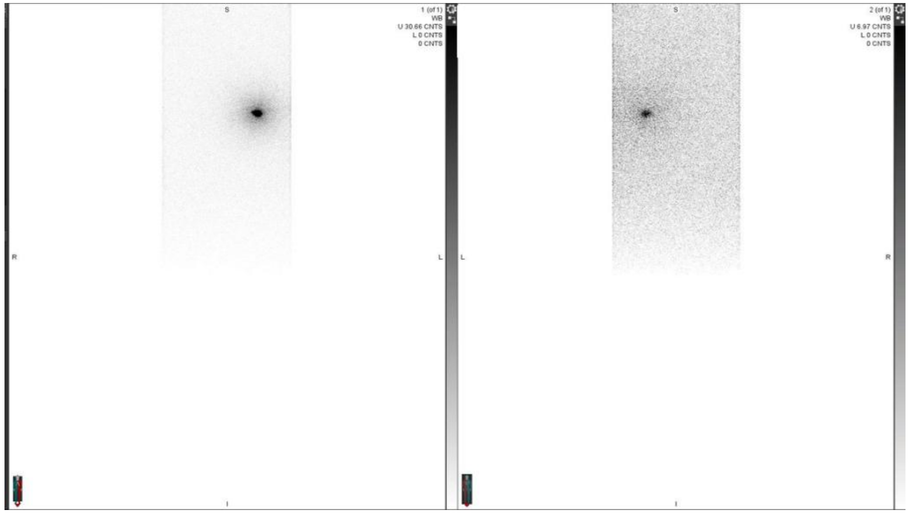

a)

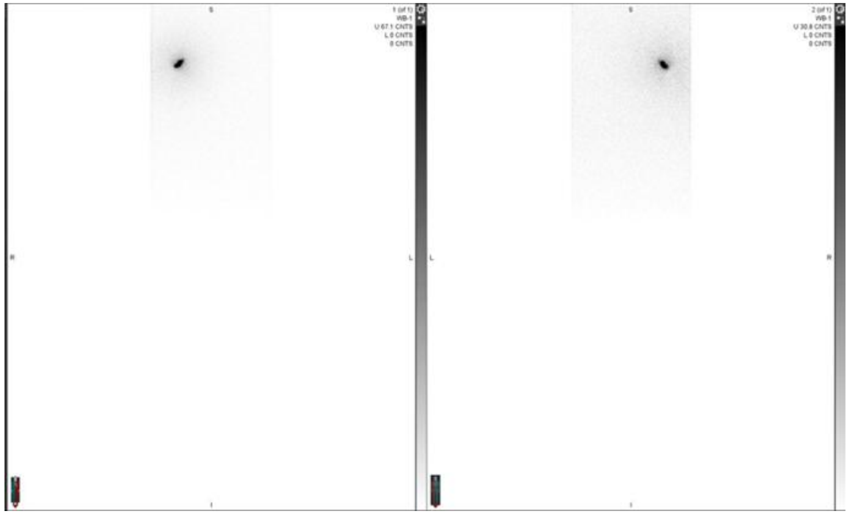

b)

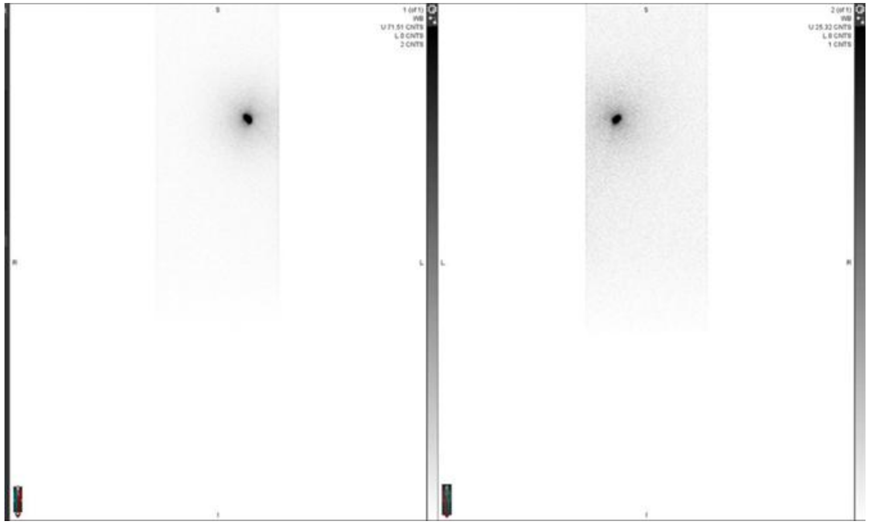

c)

Supplementary Figure S1. Representative whole-body bremsstrahlung images after intralesional [^{90}Y]Y-DOTA-biotin administration. Anterior and posterior planar whole-body images from representative patients treated at (A) 28 MBq, (B) 57 MBq, and (C) 126 MBq nominal activity levels. Qualitative assessment shows focal activity at the breast injection site without visually apparent clinically relevant extra-mammary localization. Representative whole-body bremsstrahlung images after intralesional [^{90}Y]Y-DOTA-biotin administration. Anterior and posterior planar whole-body images from representative patients treated at (A) 28 MBq, (B)

57 MBq, and (C) 126 MBq nominal activity levels. Qualitative assessment shows focal activity at the breast injection site without visually apparent clinically relevant extra-mammary localization.
